# Nutrition and Gut Microbiome in the Prevention of Food Allergy

**DOI:** 10.3390/nu17213320

**Published:** 2025-10-22

**Authors:** Mohammad Aminullah Nurain Binti, János Tamás Varga

**Affiliations:** Department of Pulmonology, Semmelweis University, H-1083 Budapest, Hungary; nurain.mohammad@stud.semmelweis.hu

**Keywords:** gut microbiome, nutrition, prebiotics and probiotics, diet, immunomodulatory metabolites, IgE-mediated food allergy

## Abstract

**Background:** Food allergies are increasingly recognized as a global health concern, influenced by early-life nutrition and the gut microbiome. This systematic review examined randomized controlled trials from 2005 to 2025 assessing the effects of probiotics, prebiotics, and synbiotics in preventing food allergies. **Methods:** Fourteen studies involving 5685 participants, including pregnant women, infants, and children with or without diagnosed food allergies, were analyzed. While several interventions demonstrated modulation of gut microbiota and immune responses, most trials reported no statistically significant reduction in IgE-mediated food allergy compared with placebo. **Results:** Some evidence suggested benefits from early exposure to allergenic foods and specific probiotic strains, such as *Lactobacillus rhamnosus* GG, particularly in cow’s milk allergy. However, heterogeneity in study designs, strains, dosages, and diagnostic criteria limited generalizability. **Conclusions:** Overall, microbiome-targeted nutritional interventions show biological plausibility but inconsistent clinical efficacy. Future large-scale, standardized, and mechanistic studies integrating microbiome, genetic, and environmental data are warranted to define optimal strategies for allergy prevention.

## 1. Introduction

A food allergy (FA) is a pathogenic immunological response triggered by consuming an antigen found in food proteins. Minimal exposure to allergic foods can cause clinical symptoms, such as urticaria, lung irritation, and gastrointestinal problems, that range in intensity from moderate to life-threatening [1]. Food allergies (FA) can be classified as either immunoglobulin E (IgE)-mediated or non-IgE-mediated, depending on the role of IgE in their development [2]. According to recent reviews of studies published in the past few years, food allergies affect approximately 8% of children and 10% of adults in developed countries, representing a significant public health issue. While the global prevalence of food allergies differs across regions, environmental factors potentially interacting with genetic susceptibility are considered the main contributors to their rising incidence [3]. In recent decades, both the prevalence of food allergies and the rate of hospitalizations due to food-induced anaphylaxis have risen significantly. This trend is often described as the “second wave of the allergy epidemic,” succeeding in the earlier surge in respiratory allergies and asthma over the past several decades [4,5,6]. Food allergens were found to be responsible for 79% of recurrent anaphylaxis cases and 37% of intensive care unit (ICU) admissions related to anaphylaxis [7]. IgE-mediated food allergies are most prevalent during infancy and early childhood, largely due to the relatively high occurrence of egg and cow’s milk allergies, which often resolve as children grow older. On the other hand, allergies to peanuts and tree nuts, which also usually appear in infancy, are less likely to go away and hence become more common in later life [8]. The most frequent allergies are fish and shellfish for adults, peanuts and tree nuts for youngsters, and milk and eggs for babies. Since milk or egg allergies in young children are likely to go away, it is critical to monitor them and reintroduce the food whenever feasible [9]. There are various ways to classify food reactions, but the two most prevalent ones are toxic and nontoxic. Nontoxic reactions can be further separated into immunological and nonimmunological reactions. The type of reaction determines the further division of immunologic responses [10].

Early life nutrition and its interaction with the gut microbiome are considered pivotal in shaping the likelihood of developing a food allergy [11,12]. The gut microbiota plays a crucial role in establishing oral tolerance, and disruptions caused by factors like Cesarean delivery, diet, or antibiotic use may impact the development of disease [13]. The gut microbiome generates metabolites, especially short-chain fatty acids (SCFAs), that connect nutrition, microbiome, and the immune system [11].

A class of beneficial, active bacteria known as probiotics can colonize the host’s gut to enhance the balance of intestinal microbes and provide positive effects [14]. Probiotics are anticipated to be used in the clinical treatment of allergic illnesses since they can improve intestinal mucus’s immunological function and preserve intestinal flora’s dynamic balance [15]. It has also been demonstrated that probiotics can lessen the allergic reactions to several food allergies. By preserving the stability and balance of intestinal flora, fortifying the intestinal barrier, and boosting the body’s metabolism, probiotics have a major impact on the capacity to increase the human body’s immune response and suppress allergies [16]. Another study indicates that probiotics can influence the immune response by enhancing pathogen elimination and modulating the adaptive immune system. When given during the perinatal period, they may help prevent allergies, although their effectiveness in allergy treatment is still uncertain [17].

Prebiotics aim to improve health by targeting the microbiota associated with humans and animals. Prebiotics are nonviable substrates that act as nourishment for beneficial bacteria that are carried by the host, such as native (resident) microorganisms, and provide probiotic strains, whereas probiotics use living microorganisms. Prebiotics are therefore distinct from most dietary fibers, including cellulose, pectins, and xylans, which promote the growth of a diverse range of gut microbes. What we mean by this is that a prebiotic should induce a metabolism skewed toward bacteria that promotes health within the native environment rather than being widely metabolized [18]. Prebiotics can indirectly influence the immune system by altering the composition and abundance of gut microbiota [19].

Synbiotics are a combination of probiotics and prebiotics used to enhance the health of humans or animals [20]. Probiotic bacteria use prebiotics as a specific growth substrate in synbiotic food products [21,22]. A group of specialists recently updated the notion of synbiotics by the International Scientific Association for Probiotics and Prebiotics. They distinguish between two kinds of synbiotics: synergistic and complementary. A probiotic and a prebiotic that work together to provide one or more health advantages without requiring co-dependent functions make up a complementary synbiotic. Co-administered microorganisms selectively use a substrate found in a synergistic symbiotic [23]. The use of probiotics and prebiotics together (synbiotics) may provide a synergistic effect in enhancing gut health [24].

This review aims to examine the impact of nutrition and the gut microbiota, including probiotics, prebiotics, and synbiotics on reducing the prevalence of food allergies. Additionally, it also focuses on summarizing the clinical trials found in the scope of the food allergy test study.

## 2. Methods

### 2.1. Search Strategy and Study Selection

**Figure 1:**
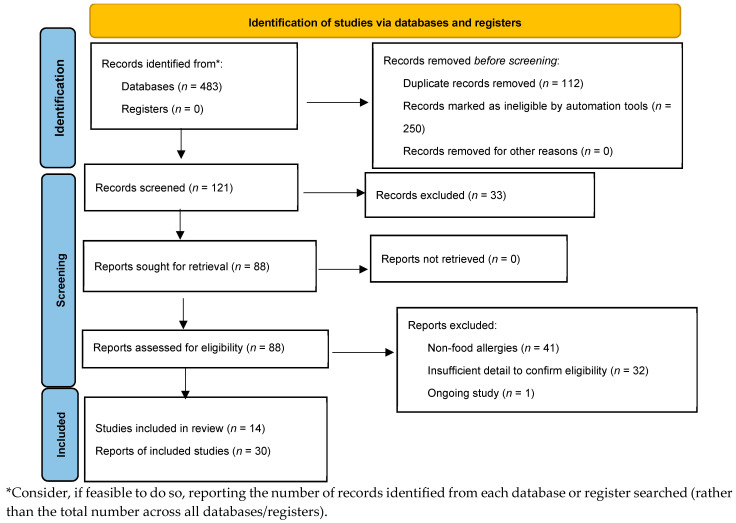
Preferred Reporting Items for Systematic Reviews and Meta-analyses (PRISMA) flow.

A comprehensive literature search was conducted in PubMed, Scopus, Multidisciplinary Digital Publishing Institute (MDPI), and Web of Science databases from 2005 until 31 March 2025. Detailed search strategies were developed for each database related to the keywords of food allergies, gut microbiome, nutrition, probiotics, prebiotics, diet, immunomodulatory metabolites, and IgE-mediated food allergies (Figure 1). We searched for randomized controlled trials (RCTs) published from 2005 to the present. Reference lists for primary research and review articles were manually reviewed for additional references, and PubMed was examined for errata or retractions related to the included studies. The review process was described using a PRISMA diagram [25].

### 2.2. Participants

This review considered studies that included participants from the neonatal period up to three years of age, as this is a critical window for immune system maturation, gut microbiome development, and the onset of food allergies. Eligible studies involved newborns, infants, and young children with or without a diagnosed food allergy, as well as studies that recruited pregnant women who consumed probiotic or prebiotic interventions during pregnancy to influence allergy outcomes in their offspring. Both maternal and child populations were therefore included to capture evidence on the role of early-life nutritional and microbiome-related interventions in the prevention or management of food allergies.

### 2.3. Types of Interventions

This review encompassed research that examined dietary and microbiome-focused strategies to prevent or treat food allergies in young children. Probiotic, prebiotic, synbiotic, or postbiotic treatments were eligible if they were given directly to newborns and young children (0–3 years old) or indirectly through maternal nutrition during pregnancy and/or lactation. If dietary changes were intended to alter immunological tolerance or the composition of the gut microbiota, supplementary feeding techniques, elimination diets, or the introduction of hydrolyzed formulas were also taken into consideration. Any form of intervention, such as capsules, powders, fortified foods, or formula milk, could be used, and it was included regardless of frequency, duration, or dosage. Excluded studies evaluated vaccinations, non-nutritional interventions, or pharmaceutical treatments.

### 2.4. Risk of Bias

**Figure 2:**
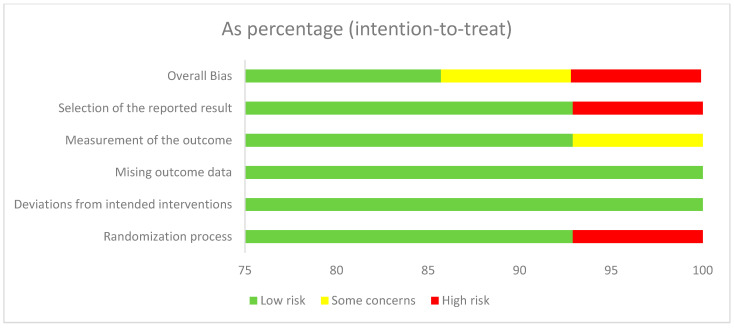
Risk of bias graph.

The RoB2 tool summary and graph results are shown in Figure 2. One report shows “high risk” for overall bias and selection of the reported results. Two studies reported “some concerns” for overall bias and measurement of the outcome. Another report on issues of “high risk” with the randomization process involved only one study. The review process involved three independent reviewers, each of whom conducted their evaluations independently to minimize bias.

## 3. Prevalence of Food Allergies

Food allergies (FA), chronic immune-mediated illnesses, have been found to affect individuals of different ages, socioeconomic backgrounds, and ethnicities [26,27]. Due to the noticeable increase in incidence and healthcare use over recent decades across many regions, food allergies have emerged as a major global concern [28].

A recent systematic review reported that in Europe, the point prevalence and lifetime prevalence of self-reported food allergies are 13.1% and 19.9%, respectively. By comparison, prevalence based on skin prick tests is 5.7%, oral food challenges (OFC) show 0.8%, and assessments using specific IgE (sIgE) levels indicate 16.6% [29]. Another recent systematic review and meta-analysis found that the prevalence of the eight most common food allergies in Europe did not differ between the periods 2000–2012 and 2012–2021 [30]. A 2018 US population-based cross-sectional survey estimated that about 1 in 13 children had at least one active IgE-mediated food allergy, consistent with previous estimates from a similar-sized US parent-report survey [31,32]. The survey also estimated that 10.8% (95% CI, 10.4–11.1%) of US adults had a confirmed food allergy, although 19.0% (95% CI, 18.5–19.5%) self-reported having one, and 5.1% had both a confirmed food allergy and a corresponding physician diagnosis for at least one allergy [28].

Although it is challenging to make definitive conclusions on trends in Asia because of the scarcity of large population-based epidemiological surveys, these trends seem to differ significantly from those of Western populations [33]. Children from five cities in China (Hong Kong and Guangzhou), Russia (Tomsk), India (Bengaluru and Mysore), and a rural county in Southern China (Shaoguan) took part in a multicenter epidemiological study using the EuroPrevall screening questionnaire. Overall, food allergy rates were low, with Hong Kong showing the highest prevalence of probable food allergy at 1.50%, followed by Russia at 0.87%, Shaoguan at 0.69%, Guangzhou at 0.21%, and India at 0.14% [34]. Both sensitization and food allergy were significantly higher in children born and raised in Hong Kong compared to those who immigrated from mainland China. This finding underscores the crucial role of early-life exposures in shaping the development of food sensitization and allergies later in life. Similarly, compared to other countries, the Indian population shows a notably lower prevalence of food allergies (1.2%) but a higher rate of food sensitization (26.5%) [35].

In Africa, the health burden of food allergies remains poorly characterized, with only a few notable studies [36,37,38]. For example, a prospective observational study at a pediatric university hospital in Cape Town reported a high overall prevalence of food sensitization (66%) and food allergy (40%), most commonly to egg (25%) and peanut (24%) [39]. The study also identified severe eczema, age under 2 years, and early-onset atopic dermatitis (before 6 months) as significant risk factors for food allergy. The unexpectedly high prevalence of food allergies observed prompted the initiation of the South African Food Sensitization and Food Allergy (SAFFA) study, a larger investigation conducted in an unselected pediatric population [40].

## 4. Intestinal Permeability, Gut Microbiota, and Their Roles in Food Allergies

Due to its possible involvement in the development of FA, leaky guts have attracted a lot of attention. According to the “epithelial barrier hypothesis”, the development of allergy disorders and sensitization can be attributed to intestinal barrier malfunction, which heightens vulnerability to environmental stimuli [41]. Intestinal integrity changes, which are frequently linked to leaky guts, may contribute to the observed increase in FA prevalence, according to this theory [42]. Industrialization and the use of ultra-processed foods (UPFs) are two examples of environmental and lifestyle factors that are thought to contribute to the disruption of intestinal barrier integrity [41]. These outside variables, which make up the external exposure, can affect the gut microbiota and epithelial barriers, which can have a major impact on the emergence of allergy disorders [43]. The integrity of intestinal epithelium is negatively impacted by food emulsifiers found in UPFs, such as polysorbate 20 and 80 [44]. It is believed that lower vitamin and antioxidant levels in UPFs make people more vulnerable to allergic reactions [43]. Additionally, it has been suggested that the higher incidence of FA may be related to heightened exposure to advanced glycation end products (AGEs) [45].

When the epithelial barrier is breached, the immune system releases inflammatory mediators called alarmins [46]. Alarmins, including TSLP, IL-33, and IL-25, are cytokines produced by epithelial cells in response to cellular damage induced by stress or infection [47,48,49]. While these cytokines play important functions in gut epithelial homeostasis, they can also create a pro-allergic microenvironment by activating T helper 2 and type 2 innate lymphocytes [50,51,52,53,54,55,56,57].

Allergic compounds, including food proteins, toxins, and microbial metabolites, can pass through the intestinal epithelium and interact with immune cells, leading to gut-associated lymphoid tissue [58]. An abnormal immunological response triggers the creation of allergen-specific IgE antibodies, which activate mast cells and basophils. When exposed to an allergen again, IgE antibodies attach to mast cells and basophils, causing the release of proteases and inflammatory mediators such as histamine [49,59]. The immune response affects intestinal permeability, leading to increased allergen transit and subsequent hypersensitive reactions. Allergies can compromise the mucosal barrier, leading to increased intestinal permeability [60,61,62].

Interactions between microbiota and hosts are critical for immune system regulation. The gut microbiota may play a role in FA development by modulating food allergy tolerance [63]. The gut microbiome increases tolerance by activating ROR-γt+ regulatory T-cells, which can be assisted by microbial synthesis of short-chain fatty acids like butyrate [64]. The intestinal microbiota can decrease allergy sensitization to food antigens by activating immune cells to produce IL-22. This improves intestinal epithelial integrity and reduces immune system contact with allergens [65].

## 5. The Effects of the Gut Microbiome

The collection of microorganisms and their genetic components inside a certain habitat is known as the microbiome. The importance of microbiomes to human physiology has become evident. The microbiome is believed to mediate health and disease, much like gene variations in the human genome [66]. Interactions between the microbiota and the host are critical to immune system modulation. While the use of antibiotics can disrupt gut homeostasis and drastically raise the risk of allergic disorders, early life exposure to maternal bacteria through vaginal or natural delivery and breast milk greatly promotes the establishment of a healthy gut microbiota and immune system [67].

The gut microbiota is a key player in the intricate sensitization system. Since cesarean babies do not inherit their mother’s vaginal microbiome, they are known to be more susceptible to allergy disorders. Additionally, low microbial diversity and an increased *Enterobacteriaceae/Bacteroidaceae* ratio are linked to later food sensitization in children. Furthermore, short-chain fatty acids (SCFAs), which are metabolites of bacteria, are also implicated. SCFAs are byproducts of bacterial fermentation, primarily from *Firmicutes*, of which *butyrate* and *propionate* are the most important. The anti-inflammatory properties of these molecules enhance the integrity of the epithelial barrier and lessen the likelihood of sensitization to dietary allergies [68].

Dysbiosis, a disorder that disrupts the original microbiota composition, causes food allergies [68]. The disruption of the gut microbiota and decreased parasite infection rates brought about by improved hygiene practices have led to a Th2-biased immune response in response to harmless stimuli like food or pollens, as well as a drop in RORγt+ Treg cells of IL10-producing regulatory B cells [69,70,71]. Another reason for the conflicting association between food allergies and the hygiene hypothesis was presented in a recent publication. They discovered that the parasitic flatworm *Schistosoma* can produce the “blocking antibody” IgG, which can cross-react with allergens like Ara h 1 and prevent allergic hypersensitivity reactions [72]. Another chemical that the parasite secretes, TGF-β, could activate Foxp3+ Treg cells. In allergic reactions, they may work together to inhibit the FcεRI on mast cells and counteract IgE [73].

Recent study on the gut microbiota of persons with food allergies has utilized *16S rRNA* gene sequencing, which encodes for a prokaryotic ribosome component. The *16S rRNA* gene enables more extensive bacterial identification without the restrictions of culture-based approaches, since it incorporates hypervariable regions and highly conserved primer binding sites, which can produce species-specific signature sequences [74]. Gut dysbiosis may occur before food allergies develop, according to findings from well-defined *16S rRNA* sequencing studies of food sensitivity and allergies. Sensitization (sIgE < 0.10 kUA/L) to at least one dietary allergy among milk, egg, peanut, soy, and wheat was connected to lower relative abundances of *Haemophilus*, *Dialister*, *Dora*, and *Clostridium* in stool samples taken from 225 US children aged 3 to 6 months. Furthermore, in stool samples taken between the ages of three and six months, the researchers discovered decreased relative abundances of *Citrobacter*, *Oscillospira, Lactococcus*, and *Dorea* in children who, by the age of three years, had a food allergy, as indicated by sensitization and perceptible allergic symptoms [75].

The role of microbiomes in food allergy has been better understood by murine models, which have yielded interesting experimental insights. These models indicate that gut microbiota may influence the risk of food allergies. In contrast to wild-type mice, mice with a gain-of-function mutation in the IL-4 receptor-α chain that are prone to food allergies have distinct intestinal abundances of *Lachnospiraceae*, *Lactobacillaceae*, *Rikenallaceae*, and *Porphyromononadaceae*. It appeared that illness vulnerability was transferred when the gut microbiota of these food allergy-prone mice was transferred to germ-free mice. Reconstituted mice showed OVA-specific IgE responses and symptoms resembling anaphylaxis upon OVA challenge. It is interesting to note that while treating these food allergy-prone mice with Treg cells specific to the immunodominant OVA peptide reduced allergen sensitization and anaphylaxis correlations, the gut microbiota did not return to normal, indicating that immunomodulatory mechanisms other than Treg cells are involved [76].

The gut microbiome has the potential to alter basophil populations, which can therefore influence allergic reactions. Mice given broad-spectrum antibiotics showed increased numbers of steady-state circulating basophil populations, improved Th2 cell responses, and higher IgE concentrations [77]. Basophil removal in these mice lowered Th2 cell responses, implying that basophils had a role in the observed allergic inflammation. Microbe-generated signals have also been identified to regulate the proliferation of bone-marrow resident precursor populations for basophil development [77].

The discovery that the gut microbiota can influence a person’s susceptibility to food allergies opens the prospect of therapeutic benefits from modifying the gut microbiota for the host’s benefit. Given the potential benefits and opportunities to impact the development and treatment of food allergies, this topic is of tremendous interest [78].

## 6. Effects of Nutrition on Food Allergies

Dietary interventions, along with the use of probiotics, prebiotics, synbiotics, and fecal microbiota transplantation, can effectively alter the gut microbiome [78]. The following table lists the impact of probiotic strains on food allergies (Table 1).

Probiotic use is often intended to alter the intestinal microbiota’s composition and activity [15,87]. The discovery that allergic children have a unique microbiota composition supports the case for altering the gut microbiota in the case of allergies [88,89]. When given in sufficient quantities, probiotics are best described as live microorganisms that, when administered in adequate amounts, confer a health benefit on the host [90]. The two primary bacterial genera that make up most probiotics are *Lactobacillus* and *Bifidobacterium*. These probiotics provide several advantages for the immune system and gastrointestinal tract, such as avoiding autoimmune disorders, allergies, and infections [91].

In a double-blind, randomized, placebo-controlled trial (DBPCRT), *Lactobacillus rhamnosus* GG (LGG) was administered to mothers who had at least one first-degree relative with an atopic disorder during pregnancy and to their infants for six months following delivery. At two years of age, the probiotic group had half the number of atopic eczema cases as the placebo group. At that time, LGG looked to be successful in preventing early atopic disease in high-risk children [92].

Probiotic supplementation with *Lactobacillus casei* and *Bifidobacterium lactis* in children with milk allergies for a year did not improve the remission of their milk allergy, according to clinical investigations [93,94]. After 6 and 12 months, however, a different study of children with milk allergies who were supplemented with *Lactobacillus rhamnosus* GG and extensively hydrolyzed casein formula showed higher rates of milk allergy resolution than a control group that was given the formula alone [93,94]. The absolute risk difference between the experimental arms in terms of cow’s milk tolerance at 12 months was 0.20 (95% CI 0.05–0.35). Researchers compared stool samples from healthy and cow’s milk-allergic infants both before and after they received extensively hydrolyzed formula, with or without *Lactobacillus rhamnosus* GG. They suggested that the presence of certain bacterial strains is associated with tolerance, as tolerant infants exhibited greater abundance of *Blautia* and *Roseburia* species and higher butyrate concentrations in their gut microbiome [95].

Research has also explored the effects of *Lactobacillus rhamnosus* GG on peanut allergy, based on mechanisms comparable to its proposed influence on other food allergies. In an experimental study, participants received peanut oral immunotherapy supplemented with *Lactobacillus rhamnosus* GG for 18 months, resulting in markedly higher desensitization rates than those observed with placebo treatment (82.1% versus 3.6%) [96].

In mice with milk β-lactoglobulin allergy, *Lactobacillus paracasei* L9 was shown to have a regulatory effect on the Th1/Th2 imbalance of lymphocytes. This effect may have been related to the bacteria’s ability to increase the number of CD4+FOXOP3+Treg cells and encourage the secretion of regulatory cytokines by DCs [97,98,99]. When *Lactobacillus* triggers a mucosal immune response, it stimulates Th2 cells to generate a significant amount of IL-5. Important IgA-stimulating substances cause B lymphocytes to release IgA, which forms the antibody secretory IgA (sIgA) by covalently attaching to protein receptors made by intestinal mucosal epithelial cells [94]. Pathogenic antigens are bound by the secreted globulin antibody sIgA, which stops them from adhering to the intestinal mucosa [100].

Experiments have been conducted to determine whether *Bifidobacterium infantis* (BB) and its antioxidant enzyme superoxide dismutase (SOD) have therapeutic effects on FA OVA-sensitized mouse models. It was shown that giving BB orally dramatically decreased the amounts of OVA-specific IgE and IgG1 in the serum and splenocytes of allergic mice, along with the release of IL-4, IL-5, and IL-13. Additionally, BB therapy reduced anaphylactic symptoms. BB enhanced the levels of glutathione (GSH) and SOD in dendritic cells (DCs), lowered reactive oxygen species (ROS) and malondialdehyde (MDA), and decreased oxidative stress in DCs. The explanation is that BB suppressed the production of TIM4, a receptor that facilitates TH2 responses and mediates DC absorption of apoptotic cells. By decreasing its phosphorylation and nuclear translocation, BB also inhibited the activation of STAT6, a transcription factor that controls the production of TIM4 [101].

According to findings from earlier gut microbiota research, probiotic benefits are probably strain-specific, and therefore careful consideration of strain-level effects and interventions is merited [78]. Probiotics have been shown to increase Th1 and regulatory cytokine production in vitro, but their ambiguous and unpredictable immunomodulatory effect in vivo is debatable [102]. According to this viewpoint, the data supporting the use of probiotics to treat FA seems debatable. There are currently no clear guidelines about which strain to use, at what dosage, and for how long, even though cow milk allergy (CMA) seems to be the most researched type of FA [103].

### 6.1. Mechanism of Probiotics in Gut Modulation

According to Mazziotta et al. [104], probiotic bacteria that are consumed attach themselves to intestinal epithelial cells and use pattern recognition receptors (PRRs) to activate them. Probiotic-stimulated cytokines activate T regulatory (Treg) cells, which preserve immunological homeostasis in the gut mucosa. Tregs are important in reducing the immune response because they are powerful immune response suppressors. Tregs induction results in the release of transforming growth factor (TGF)-β or IL-10. Furthermore, probiotics cause B cells to mature into plasma cells that produce immunoglobulin (Ig)A. Cytokine and chemokine release from intestinal epithelial cells creates a microenvironment in the intestinal lamina propria that enables B cells to proliferate clonally and make IgAs. IgAs regulate bacterial adherence to the host tissue after migrating past the epithelium and into the mucus layer [104].

### 6.2. Prebiotics

Typically, linked carbohydrates like oligosaccharides and short-chain polysaccharides make up prebiotics. The molecular property of these molecules is that they cannot be broken down by the intestinal tract’s enzymes. As a result, they can either directly interact with the surrounding cells or act as food sources for microbes deemed “beneficial,” such as *Bacteroides* and *Bifidobacterium* [105].

Distinct prebiotics encourage the proliferation of particular native gut microbial populations. While the resulting strain- and species-level changes are often unpredictable, prebiotics hold great promise for influencing the gut microbiome. The gut environment, especially factors such as pH, strongly affects the dynamics of interspecies interactions. Consequently, personalized microbial profiles should be carefully considered during the development of prebiotics to maximize effectiveness and minimize safety risks [106]. Lists of relevant studies is added in the table of effects of prebiotics (Table 2).

Non-digestible food ingredients, known as prebiotics, specifically enhance the proliferation and metabolic activity of the host’s commensal microbiota. Fiber, a common prebiotic, is utilized by gut bacteria to generate short-chain fatty acids, which are believed to contribute to the reduction in allergic inflammation [120,121]. Prebiotics are often added to infant formulas. Those that remain intact through the upper gastrointestinal tract reach the large intestine, where they are specifically metabolized by beneficial microorganisms [122]. Prebiotic nutrition in babies has not been found to have any impact on the onset of food allergies in clinical trials [122,123,124]. However, in certain individual investigations, the risk of eczema and asthma decreased [122,123,124].

An excellent illustration is the prebiotic Bimuno (Clasado Biosciences Ltd., Reading, UK). In terms of GOS, it is a lactose-based mixture made with probiotic *Bifidobacterium bifidum* NCIMB 41171 enzymes. It can directly interact with the immune system to enhance the gut’s barrier function. In overweight people with metabolic syndrome, its beneficial immunomodulatory effect was further evidenced by a considerable rise in gut immunological parameters that protect against pathogens and a decrease in inflammatory markers in the blood and feces [125].

Prebiotics, known as human milk oligosaccharides (HMOs), are found in human milk. Oligosaccharides (5 g/L to 23 g/L) with a lactose-reducing end extended with fucosylated and/or sialylated *N*-acetyllactosamine units make up human milk and colostrum. The size, charge, and order of the more than 150 HMO configurations vary [126]. Neutral fucosylated and non-fucosylated oligosaccharides are the most common HMOs [127]. HMOs are minimally absorbed through the intestinal wall and provide no direct nutritional value to the newborn [128]. Rather, it is proposed that HMOs can serve the infant in a variety of additional capacities. They serve as prebiotics, encouraging the development of advantageous intestinal flora and influencing the gut microbiome. They are also favored substrates for several gut bacterial species. The fermentation of HMOs by the gut microbiota produces short-chain fatty acids (SCFA), which are essential for intestinal health [129].

### 6.3. Synbiotics

Synbiotics function to stimulate the growth of native gut bacterial strains and to increase the survival rate of health-promoting microbes delivered through dietary sources [17]. How synbiotics influence metabolic health is not yet fully understood. Notably, the health effects of synbiotics are probably dependent on the combination of probiotics and prebiotics used [130]. The use of synbiotics to modify the human gut microbiota appears promising given the vast array of potential combinations [131].

Synbiotics, combining probiotics and prebiotics, are under investigation in clinical trials for their role in allergy prevention. In a prospective, randomized, double-blind controlled trial, 110 full-term infants with cow’s milk allergy were given either amino acid–based formula (AAF) alone or AAF with synbiotics. Both groups showed normal growth and a reduction in allergic symptoms. This is the first study to demonstrate that an AAF containing a specific synbiotic blend promotes normal growth in infants with cow’s milk allergy, comparable to that observed with AAF alone [132]. A summary of the relevant research has been listed in the table below that investigated the impact of probiotics, prebiotics, and synbiotics in managing food allergies (Table 3).

## 7. Discussion

A review of cumulative research suggests that nutrition interventions such as probiotics, prebiotics, or synbiotics may reduce the likelihood of allergic reactions. However, clinical trials have not demonstrated significant differences when compared to placebo-controlled groups. This lack of strong evidence limits the clinical relevance and practical application of these nutritional approaches in managing food allergies.

Multiple studies have found no significant differences between intervention and placebo groups regarding the prevention of or reduction in cow’s milk protein allergy (CMPA) or other food allergies. For example, research by Palmer et al. [134] indicated that prebiotic or probiotic supplementation during pregnancy and postpartum did not substantially decrease the occurrence of IgE-mediated food allergies in children and Komulainen et al. [142]. Similar findings were made by Chatchatee et al. [139] and Loke et al. [140], who reported no discernible benefit of using probiotics or synbiotics over control formulas in terms of helping allergic youngsters improve their outcomes or acquire tolerance. However, a few studies demonstrated potential benefits. Sakihara et al. [146] found that infants who drank cow’s milk formula from an early age had a much lower incidence of CMA cases than those who did not (0.8% vs. 6.8%, *p* < 0.001). In a similar vein, Skjerven et al. [145] discovered that early exposure to allergenic foods, especially in a food intervention group, decreased the occurrence of food allergies when compared to non-intervention.

Biological impacts were also demonstrated in animal model experiments. Although it is unclear how this might translate to human outcomes, Xu et al. [136] showed that high-dose probiotic supplementation dramatically decreased OVA-specific IgE levels in mice, indicating a reduction in an allergic response. A notable study by Nocerino et al. [143] revealed that, when compared to other formulas, babies treated with an extensively hydrolyzed casein formula including *Lactobacillus rhamnosus* GG (EHCF + LGG) had the lowest incidence of atopic symptoms for 36 months.

The findings of this review are consistent with the broader body of evidence showing mixed outcomes regarding the efficacy of nutritional interventions, particularly probiotics, prebiotics, and synbiotics in managing or preventing food allergies. Several studies included in this review, such as those by Cukrowska et al. [133] and Nocerino et al. [143], demonstrated improvements in allergic symptoms and immune modulation following probiotic supplementation, particularly with *Lactobacillus rhamnosus* GG and related strains. However, other studies (Palmer et al. [134]; Yamamoto-Hanada et al. [138]; Komulainen et al. [142]) found no statistically significant effects, mirroring the inconsistency frequently reported in the wider literature.

Recent meta-analyses have similarly underscored the variability in outcomes. A 2024 systematic review reported that probiotics are likely to have no significant effect on reducing eczema scores among infants with cow’s milk allergy. However, modest improvements may occur in children with other allergic conditions [147]. Another comprehensive review emphasized that combined maternal and infant supplementation produced greater preventive benefits than supplementation during only one life stage, highlighting the importance of timing and developmental context [148]. Conversely, earlier meta-analyses of Tang et al. [149] and Cuello-Garcia et al. [150] concluded that prenatal or postnatal probiotic use did not significantly prevent food allergies overall, though modest risk reduction was observed for atopy or sensitization outcomes [149,150].

In 2012, the World Allergy Organization’s (WAO) Special Committee on Food Allergy and Nutrition conducted a qualitative and narrative assessment of probiotic treatment for allergic diseases. The scientists found that probiotics do not have an established role in allergy prevention or treatment. There is no evidence that a single probiotic pill or class of nutrients can significantly impact allergy symptoms or long-term disease progression [151]. A few years later, the WAO organized a panel of guidelines to create evidence-based suggestions for using probiotics to avoid allergies. Using a GRADE approach, they declared that there is currently no proof that probiotic supplementation lowers a child’s allergy. The WAO guideline panel concluded that there is a probable advantage to utilizing probiotics, primarily from the prevention of eczema, after considering all important outcomes [152].

Since bacterial microbiota has been the focus of most recent studies on microbiome in food allergies, future studies could evaluate the virome and microbiome’s contributions. Analytical tools, reference databases, and sequencing techniques for evaluating the microbiome and virome are less advanced than those for the bacterial microbiome [153]. However, research on microbiomes beyond the bacterial microbiome is becoming possible due to the growing availability of tools for the analysis of data from shotgun metagenomic sequencing, which is whole-genome sequencing done on genomic DNA from a mixed microbial population [154,155]. Studies of microbiomes, as well as data acquired from genome-wide association, have increased our understanding of food allergies [156,157,158,159]. Integrating system-wide data is crucial for constructing predictive models of complex biological interactions and systems, leading to a more comprehensive knowledge of food allergies [88,154,160,161].

Even though most human clinical trials show only slight or no statistically significant improvements in allergy prevention from probiotics, prebiotics, or synbiotics, some research suggests that certain interventions (such as early allergen exposure or specific probiotic strains) may be beneficial in preventing allergies. Administration of *Lactobacillus rhamnosus* GG is likely to promote tolerance in infants with suspected cow’s milk allergy. Only studies focusing on CMA were included, as no research was identified on the use of probiotics for treating other types of food allergies in children [103]. The only probiotic strain with the most robust and reliable RCT data for avoiding or enhancing some allergic outcomes is *Lactobacillus rhamnosus* GG (LGG), particularly in early-life allergy prevention trials and in newborns with cow’s milk allergy. This advantage, however, is strain-specific and primarily shown in children rather than adults; there is conflicting data regarding other strains or general allergy prevention. Results vary widely, most likely due to variations in populations, strains utilized, durations, and allergy criteria.

## 8. Study Limitations

This systematic review has a few limitations that should be noted. First, the included studies had significant variation in terms of participant age groups, interventions (kind, strain, and dose of probiotics, prebiotics, or synbiotics), duration of supplementation, and outcome measures, making direct comparison and pooling of results difficult. This heterogeneity can complicate the synthesis of data and may limit the ability to draw definitive conclusions from the review. Many trials had limited sample sizes and short follow-up periods, making it difficult to determine long-term effects on allergy avoidance. Furthermore, most of the research was conducted in high-income nations, which may limit the findings’ applicability to more diverse populations with varying genetic, environmental, and nutritional exposures. This can result in a limited representation of the available evidence, potentially impacting the generalizability of the review findings on different populations or settings. The use of different diagnostic criteria for food allergy among studies may have resulted in variability in reported outcomes.

## 9. Conclusions

Nutritional treatments like probiotics, prebiotics, and synbiotics offer molecular plausibility but variable clinical effects, highlighting the gut microbiome’s crucial but complex role in preventing food allergies. When compared to placebo, most randomized controlled trials show modest or non-significant results, despite some studies showing benefits from strains or early allergen exposure. Standardized preventative guidelines are challenging to produce because of these disparities, which most likely reflect variation in study designs, populations, intervention timing, and definitions of allergy outcomes. Therefore, the information now available indicates that while microbiome-modulating techniques have potential, they cannot yet be considered as effective preventive approaches.

Thorough, extensive, and long-term studies that combine microbiome analysis with genetic and environmental factors that influence allergy risk should be given top priority in future research. The identification of strain-specific effects, the best times for interventions, and synergistic dietary strategies should receive special attention. The development of focused, individualized tactics may be guided by a more thorough understanding of host–microbiome–immune interactions made possible by advancements in multi-omics and systems biology. Nutritional control of the gut microbiota should not be the mainstay of allergy prophylaxis until such evidence is consolidated; rather, it should be an auxiliary field of study.

## Figures and Tables

**Table 1:** Probiotic effects on food allergies.

Probiotic Strain	Experiment Model	Reported Impact on Food Allergy	Reference
*Lactobacillus rhamnosus* GG (LGG)	Children	Increased rate of tolerance acquisition in infants with cow’s milk allergy; LGG-supplemented formula may promote faster clinical tolerance.	[79]
*Bifidobacterium breve* M-16V	Animal (mice)	Ameliorates cow’s milk allergy–like responses and modulates gut microbiota via AhR signaling.	[80]
*Bifidobacterium longum* (KACC 91563)	Animal (mice)	Induces mast-cell apoptosis and reduces allergic responses in murine models.	[81]
*Lactobacillus plantarum* (A56)	Animal (mice)	Reduces symptom severity in ovalbumin-induced allergy models; improves immune modulation.	[82]
*Lactobacillus acidophilus* (L-92)	Children	Adjunctive therapy in atopic dermatitis with food allergy; immunomodulatory benefits noted.	[83]
*Lactobacillus casei* (various strains)	Animal (mice); children	Fermentation reduces milk protein antigenicity; strain-dependent clinical benefits.	[84,85]
*Lacticaseibacillus paracasei* (AH2)	Animal (mice)	Alleviates wheat/gluten-related allergy in animal models; modulates SCFAs and microbiota.	[86]

**Table 2:** A summary of studies that have investigated the effects of prebiotics.

Prebiotic Compound	Type/Source	Setting Studied	Key Findings	References
Galacto-oligosaccharides (GOS)	Synthetic/milk-derived oligosaccharides	Infants at risk of allergy; cow’s-milk allergy (CMA)	Reduced eczema incidence, promoted bifidobacteria, may lower allergic sensitization risk.	[107,108,109]
Fructo-oligosaccharides (FOS)	Plant-derived (inulin-type)	Infants with or at risk of allergy; food-allergic animal models	Combined GOS/FOS mixture modulated gut microbiota and immune markers, reducing atopic dermatitis and possibly CMA risk.	[107,108,109,110]
Short-chain fructo-oligosaccharides (scFOS)	Chicory/inulin-derived	Cow’s-milk allergic infants; preventive trials	scGOS/scFOS mixture enhanced immune tolerance and increased bifidobacteria counts; reduced allergic manifestations.	[109,110,111]
Human milk oligosaccharides (HMOs)	Natural prebiotics in human milk (2′-FL, LNnT, etc.)	Infants; preventive and therapeutic models of food allergy	HMOs shape immune tolerance, gut barrier function, and microbiota; emerging clinical evidence for allergy prevention.	[112,113,114]
Inulin	Plant-derived polysaccharide (e.g., chicory root)	Mouse models of food allergy (OVA, peanut)	Reduced Th2 cytokines, enhanced Treg induction, increased SCFA levels, mitigated allergic reactions.	[115,116]
Xylo-oligosaccharides (XOS)	Hemicellulose-derived oligosaccharides	Animal models of peanut and OVA allergy	Enhanced butyrate-producing bacteria and mucosal tolerance markers; potential anti-allergic benefit.	[117,118]
Lactulose	Synthetic disaccharide (galactose + fructose)	Preclinical models	Improved intestinal barrier, increased SCFAs, reduced allergic sensitization markers.	[119]

**Table 3:** Summary of the impact of nutrition on food allergies.

First Author, Year	Study Designs	Participants	Durations of Interventions	Interventions	Outcomes
Bozena Cukrowska, 2021 [133]	Multicenter, randomized, double-blind, placebo-controlled	Children under 2 years of age with AD and CMPA (*n* = 101)	3-month interventions 9-month follow-up	50% (*Lactobacillus casei* ŁOCK 0919), 25% (*Lactobacillus rhamnosus* ŁOCK 0908), 25% (*Lactobacillus rhamnosus* ŁOCK 0900)	Reduction in both groups. No significant difference. Probiotic: (−22.8 to −28.8) Placebo: (−16.7 to −23.2) *p*-value = 0.704
Debra J. Palmer, 2025 [134]	Randomized, double-blinded	Pregnant women (*n* = 652)	18–20 weeks of pregnancy until 6 months after delivery	Prebiotics for interventions: galacto-oligosaccharides (GOS) and fructo-oligosaccharides (FOS) Placebo: maltodextrin	No significant differences between the groups in the percentage of infants with IgE- mediated food allergy 1.08 (0.65 to 1.79) *p* = 0.7
Anna Nowak-Wegrzyn, 2019 [135]	Randomized, double-blind, placebo- control	CMPA-documented infants and children aged 2 months to 4 years (*n* = 61)	3–28 days (first challenge) 2–7 days (second challenge)	Whey-based EHF (Test formula) with lacto-*N*-neotetraose (LNnT) and 20 fucosyl-lactose (20FL)	95% CI lower bound: (1) Test formula = 92.8% (2) Control formula = 92.6%
Hanxue Xu, 2024 [136]	Randomized- controlled trial	BALB/c female mice, 6–8 weeks old (*n* = 40)	22 days	In allergic mice induced by ovalbumin (OVA), *Lactobacillus kefiranofaciens* ZW3	IgE levels that were specific to OVA dropped significantly (*p* < 0.01) in the LGG and high-dose ZW3 groups in contrast to the group using the food allergy model
Pingping Zhu, 2025 [137]	Multicenter, randomized, double-blind, controlled clinical study	CMA- allergic infants (*n* = 39)	12 months	AAF and AAF-S	The changes in Bifidobacterium were positively correlated with those of adenine at TP1 and TP2 in both groups (*r* > 0.5, *p* < 0.05). In the AAF-S group, changes in ILA and 4-OH- PLA# from TP0 to later time points were positively correlated with those of *Bifidobacterium* (*r* > 0.6, *p* < 0.005)
K. Yamamoto-Hanada, 2023 [138]	Double-blind, randomized, placebo-controlled	IgE-mediated CMA in children aged 1–18 years (*n* = 60)	24 weeks	Citrus juice fermented with *Lactiplantibacillus plantarum* YIT 0132 (LP0132)	Primary outcome: no significant difference in threshold CM dose (*p* = 1.00)
Pantipa Chatchatee, 2022 [139]	Multicenter, prospective, randomized, double-blind, controlled clinical study	Aged ≤13 months with IgE-mediated CMA (*n* = 169)	12 months	Probiotic *Bifidobacterium breve* M-16V and prebiotic oligosaccharides (oligofructose, inulin) infused with amino acids and synbiotics	No significant difference in terms of development tolerance between AAF and AAF-S groups (*p* = 0.36)
Paxton Loke, 2022 [140]	Multicenter, randomized, double-blind, placebo- controlled	Children aged 1–10 years with peanut allergy (*n* = 201)	8 weeks oral immunotherapy followed by 12 months post- treatment	Peanut protein with probiotic *L rhamnosus* ATCC 53103 Probiotic placebo: maltodextrin	No significant difference between PPOIT vs. OIT group. *p*-value = 0.52 PPOIT vs. placebo, *p* < 0·0001 OIT vs. placebo, *p* < 0·0001) Both PPOIT and OIT were effective at inducing sustained unresponsiveness
Eishika Dissanayake, 2019 [141]	Factorial, randomized, non- treatment- controlled trial	From birth until 1 year with AD or food allergy old (*n* = 459)	For interventions, from birth to 6 months of age, and evaluation at 1 year of age	Fructo-oligosaccharides (0.5 g) in combination with the synbiotic *Bifidobacterium bifidum* OLB6378	The prevalence of FA at 1 year of age did not show any difference between the 4 groups. Synbiotics vs. no intervention group (*p* = 0.4778)
Miisa Komulainen, 2023 [142]	Double-blind, randomized, placebo-controlled, one-center study	Women from early pregnancy to 6 months of postpartum (*n* = 439), children at 12 months (*n* = 284) and 24 months (*n* = 264)	Pregnancy up to 6 months after delivery	Fish oil capsules (n-3 fatty acids of which are docosahexanoic acid, eicosapentaenoic acid and docosapentaenoic acid. Probiotic contained *Lacticaseibacillus rhamnosus* HN001 (formerly *Lactobacillus rhamnosus* HN001) and *Bifidobacterium animalis* ssp. *lactis* 420	There were no discernible differences among the four intervention groups. In children aged 12 and 24 months, recurrent wheezing was documented in 12% and 15% of cases, atopic eczema in 15% and 18%, and a food allergy identified by a physician in 4.5% and 9.8% of cases, respectively (*p* > 0.05).
Rita Nocerino, 2021 [143]	Cohort study	Non- breastfed infants (aged 1–12 months) with suspected IgE-mediated CMA (*n* = 365)	36 months	Probiotic *L. rhamnosus* GG (EHCF + LGG), rice hydrolyzed formula, soy formula, extensively hydrolyzed whey formula (EHWF), or amino acid- based (AAB)formulas	FA incident: Rice vs. EHCF+LGG (*p* < 0.001) Soy vs. EHCF + LGG (*p* < 0.001) EHWF vs. EHCF + LGG (*p* < 0.001) AAB vs. EHCF + LGG (*p* < 0.001)
Viljanen M, 2005 [144]	Randomized, double-blind, placebo-controlled	Infants with atopic eczema/dermatitis syndrome and food allergies (*n* = 102)	4 weeks	Lactobacillus GG (LGG) Placebo group: inert matrix material, microcrysta line cellulose	Faecal IgA levels in CMA allergy infants was higher than the placebo group (0.014)
Håvard Ove Skjerven, 2022 [145]	2 × 2 factorial, cluster-randomized trial	2697 women with 2701 pregnancies. Newborn infants (*n* = 2397)	2 years	Peanuts, cow’s milk, wheat and eggs	FA was reduced in the food intervention group compared with no food intervention group (*p* = 0.004)
Tetsuhiro Sakihara, 2021 [146]	Multicenter, open label randomized-controlled trial	Newborn within 5 days of birth until 6 months of age (*n* = 491)	<5 days newborn until 6 months of age	Ingestion group: Breastfed and CMF (≥10 mL/day) Avoidance group: Breastfed supplemented with soy formula	Of the 242 participants in the ingestion group, there were 2 CMA instances (0.8%), while the 249 people in the avoidance group had 17 CMA cases (6.8%) (*p* < 0.001).

Abbreviations: AD: Atopic Dermatitis; CMPA: Cow Milk Protein Allergy; EHF: Extensively Hydrolyzed Formula; CI: Confidence Interval; BALB/c: Bagg Albino c strain of mice; LGG: Lactobacillus Rhamnosus; CMA: Cow Milk Allergy; AAF: Amino Acid-Based Formula; AAF-S: Amino Acid-Based Formula with Synbiotics; PPOIT: Probiotic and Peanut Oral Immunotherapy; OIT: Oral Immunotherapy; EHCF: Extensively Hydrolyzed Casein Formula; EHWF: Extensively Hydrolyzed Whey Formula; CMF: Cow’s Milk Formula.

## Data Availability

Data sharing does not apply to this article, as no new data were created or analyzed in this study.
